# Topical Application of Trisodium Ascorbyl 6-Palmitate 2-Phosphate Actively Supplies Ascorbate to Skin Cells in an Ascorbate Transporter-Independent Manner

**DOI:** 10.3390/nu9070645

**Published:** 2017-06-22

**Authors:** Shuichi Shibuya, Ikuyo Sakaguchi, Shintaro Ito, Eiko Kato, Kenji Watanabe, Naotaka Izuo, Takahiko Shimizu

**Affiliations:** 1 Department of Advanced Aging Medicine, Chiba University Graduate School of Medicine, 1-8-1 Inohana, Chuo-ku, Chiba, Chiba 260-8670, Japan; s-shibuya@chiba-u.jp (S.S.); kng.wtnb@chiba-u.jp (K.W.); ntk.izuo@chiba-u.jp (N.I.); 2 Reserch & Development Division, Club Cosmetics Co., Ltd., Ikoma, Nara 630-0222, Japan; ikuyos@clubcosmetics.co.jp (I.S.); sito@clubcosmetics.co.jp (S.I.); 3 Functional Chemicals Division, Showa Denko K.K. Minato-ku, Tokyo 105-8518, Japan; kato.eiko.xhzqn@showadenko.com

**Keywords:** ascorbic acid, ascorbic acid transporter, ascorbic acid derivative, skin

## Abstract

Ascorbic acid (AA) possesses multiple beneficial functions, such as regulating collagen biosynthesis and redox balance in the skin. AA derivatives have been developed to overcome this compound’s high fragility and to assist with AA supplementation to the skin. However, how AA derivatives are transferred into cells and converted to AA in the skin remains unclear. In the present study, we showed that AA treatment failed to increase the cellular AA level in the presence of AA transporter inhibitors, indicating an AA transporter-dependent action. In contrast, torisodium ascorbyl 6-palmitate 2-phosphate (APPS) treatment significantly enhanced the cellular AA level in skin cells despite the presence of inhibitors. In ex vivo experiments, APPS treatment also increased the AA content in a human epidermis model. Interestingly, APPS was readily metabolized and converted to AA in keratinocyte lysates via an intrinsic mechanism. Furthermore, APPS markedly repressed the intracellular superoxide generation and promoted viability associated with an enhanced AA level in *Sod1*-deficient skin cells. These findings indicate that APPS effectively restores the AA level and normalizes the redox balance in skin cells in an AA transporter-independent manner. Topical treatment of APPS is a beneficial strategy for supplying AA and improving the physiology of damaged skin.

## 1. Introduction

Ascorbic acid (AA) is a major soluble vitamin distributed in the tissues of all organisms, including animals and plants. In humans, organs such as the skin contain millimolar-order levels of AA, while plasma contains relatively low levels of AA (40–60 µM) [1,2]. Environmental factors such as lifestyle and nutrients consumed in the diet regulate the physiological kinetics of AA for maintaining organ homeostasis in the body. For example, smoking and an insufficient intake of vegetables and fruits adversely affect the AA status in the human body [3].

Accumulating evidence has shown that the chemical characteristics of AA as an electron donor play an important role in redox regulation in the human body [2,4,5,6]. AA also regulates many oxidase and hydroxylase activities as a cofactor to maintain cellular metabolism [4,7]. In particular, AA is an essential cofactor for post-translational modifications by lysyl oxidase and prolyl hydroxylase in collagen formation and further enhances the transcript levels of type I and III collagen genes [5,8]. AA also interferes with pigment production by interacting with copper ions at the tyrosinase activity site and reducing dopaquinone [9]. In this context, AA supplementation has been largely used to maintain the skin function and prevent skin aging in cosmetic and supplement fields worldwide.

As AA is highly fragile and not very liposoluble, allowing it to penetrate the skin and sustain its physiological function over a long period of time is difficult. To increase the stability and liposolubility of AA, various AA derivatives have been developed for dermatological application [10,11]. A phosphate group- and long hydrophobic chain-conjugated derivative, torisodium ascorbyl 6-palmitate 2-phosphate (APPS), was also developed to increase liposolubility [12]. In clinical applications, Inui and Itami have reported that topical treatment with APPS lotion for four weeks attenuated perifollicular pigmentation in female subjects [13].

AA is incorporated into cells through two types of transporters: sodium-dependent vitamin C transporters (SVCT1 and SVCT2) and hexose transporters (GLUT1, GULT3, and GLUT4) [14]. AA and oxidized AA, known as dehydroascorbic acid (DHAA), are separately transported into the cytoplasm by SVCTs and GULTs, respectively. However, precisely how the AA derivative is transported to skin cells and converted to AA remains unclear.

In the present study, we measured the cellular intake and transporter utilization of AA or APPS to estimate the intake efficiency of AA in skin cells. We also investigated the conversion mechanism of APPS to AA in cells. Furthermore, we investigated the redox regulation by APPS treatment in skin cells associated with oxidative damage. We then discussed the potential utility of APPS in AA supplementation and proposed an ideal protocol for applying AA derivatives to skin.

## 2. Materials and Methods

### 2.1. Materials

**Figure 1:**
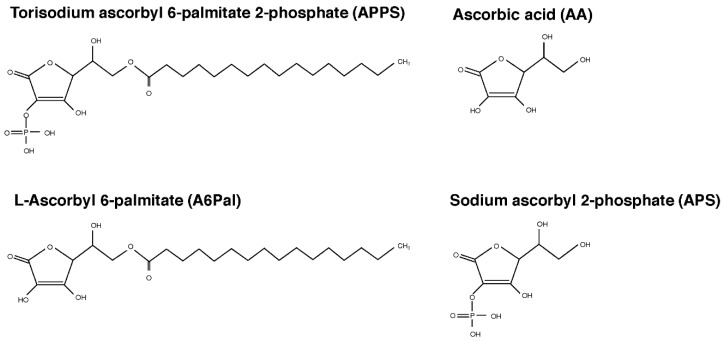
The structures of ascorbic acid (AA), A6Pal, APS, and APPS. l-ascorbyl 6-palmitate (A6Pal) is additionally conjugated with a long hydrophobic chain. Sodium ascorbyl 2-phosphate (APS) is additionally conjugated with a phosphate group. Torisodium ascorbyl 6-palmitate 2-phosphate (APPS) is additionally conjugated with a phosphate group and a long hydrophobic chain.

APPS (Figure 1) was provided by Showa Denko K.K. (Tokyo, Japan).

### 2.2. Measurement of AA Content in Skin Cells

**Figure 2:**
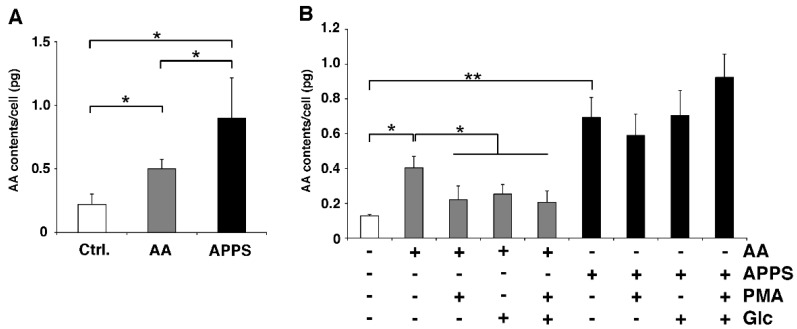
APPS upregulates the cellular AA level in an AA transporter-independent manner. (**A**) Intracellular ascorbic acid (AA) contents in human cells treated with 10 µM AA or 10 µM APPS for 1 h. These data represent the mean ± SE; * *p* < 0.05; (**B**) Intracellular AA contents in human cells. Human cells were pre-incubated with or without 10 µM PMA and 10 µM glucose for 1 h. After pre-incubation, cells were washed and cultured for 1h in culture medium with or without 10 µM AA and 10 µM APPS. These data represent the mean ± SEM; * *p* < 0.05 vs. no treatment control, ** *p* < 0.01 vs. no treatment control.

Human fibroblasts (TIG118) were purchased from Health Science Research Resources Bank (Tokyo, Japan). TIG118 cells were maintained in DMEM (Nacalai Tesque, Kyoto, Japan) supplemented with 10% FBS (Life Technologies Corporation, Carlsbad, CA, USA), 100 units/mL of penicillin (Sigma-Aldrich, St. Louis, MO, USA), and 0.1 mg/mL of streptomycin (Sigma-Aldrich) at 37 °C in a humidified incubator with 5% CO_2_. Cells were pre-incubated with or without phorbol 12-myristate 13-acetate (PMA) and glucose for 1 h to inhibit ascorbate transporters [15,16]. After pre-incubation, cells were washed three times with PBS and cultured for 1 h in culture medium with or without 10 µM AA and 10 µM APPS. Isolated cells were sonicated with 5.4% metaphosphoric acid (Wako, Osaka, Japan) to suppress oxidation. The homogenate was centrifuged at 10,000× *g* for 15 min at 4 °C, and the supernatant was then used for the assay. The AA level was measured using the Vitamin C quantitative determination Kit (SHIMA Laboratories, Tokyo, Japan) in accordance with the manufacturer’s instructions (Figure 2).

### 2.3. A Kinetic Analysis of APPS Metabolism in Vitro

**Figure 3:**
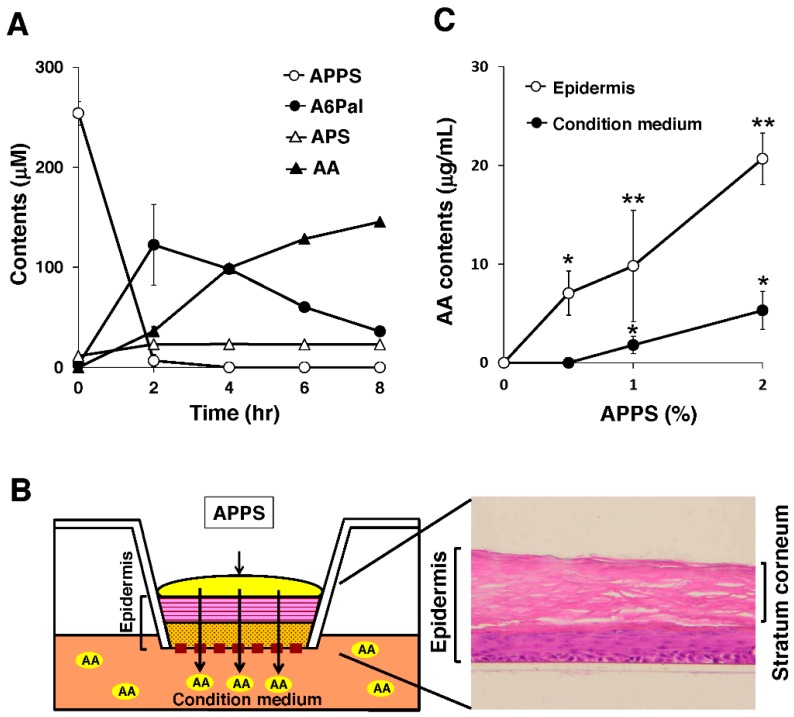
APPS is converted to AA by endogenous convertases. (**A**) A kinetics analysis of APPS metabolites including AA, A6Pal, and APS in keratinocyte lysates; (**B**) A human epidermal skin model (LabCyte EPI-MODEL) was used in ex vivo experiments; (**C**) AA contents in epidermis and conditioned medium in an ex vivo human epidermal skin model treated with APPS at various doses. These data represent the mean ± SEM; * *p* < 0.05 vs. no AA treatment, ** *p* < 0.01 vs. no AA treatment.

Human keratinocytes (NHEKs) were purchased from KURABO Industries (Osaka, Japan). NHEKs were cultured in HuMedia KG-2 (KURABO Industries) in accordance with the manufacturer’s instructions. Cultured keratinocytes were collected and homogenized with HEPES buffer (1 × 10^6^ cells/mL). To the homogenate was added 300 µM APPS (final concentration), and the solution was incubated at 37 °C. At each sampling point, the homogenate was centrifuged at 10,000× *g* for 15 min at 4 °C, and the supernatant was collected. Samples were filtered through a 0.22-µm membrane and measured for APPS and its metabolites (Figure 1) by high-performance liquid chromatography (HPLC) using a Shimadzu Prominence 20A system (Shimadzu Corporation, Kyoto, Japan). The separation conditions of AA, APS, A6Pal, and APPS were as follows, respectively: (1) for AA, Shodex Asahipak NH2P-50 4E column (Showa Denko K.K., Tokyo, Japan); detection wavelength, 254 nm; mobile phase, 60 mM H_3_PO_4_/acetonitrile (20/80); flow rate, 0.8 mL/min; (2) for APS, Shodex Asahipak NH2P-50 4E column; detection wavelength, 245 nm; mobile phase, 45 mM Na_2_SO_4_, 50 mM H_3_PO_4_/acetonitrile (80/20); flow rate, 1 mL/min; (3) for A6Pal and APPS, Shodex Silica C18P 4E column (Showa Denko K.K., Tokyo, Japan); detection wavelength, 265 nm; mobile phase, 30 mM K_2_HPO_4_ (pH 7.0)/tetrahydrofuran (35/65); flow rate, 0.7 mL/min. The levels of APPS and its metabolites were determined on the basis of the peak area of the standard AA curve (Figure 3A).

### 2.4. Treatment with APPS in a Human Epidermal Skin Model

A human epidermal skin model (LabCyte EPI-MODEL; J-TEC, Aichi, Japan) was cultured in accordance with the manufacturer’s instructions (Figure 3B). The skin model was treated with APPS solution and cultured at 37 °C for 24 h. After incubation, skin tissues and conditioned medium were collected. Skin tissues (10 mm diameter) were homogenized with 50% ethanol (three tissues/1.5 mL) using a Biomasher (Nippi, Ibaraki, Japan). The skin homogenate was centrifuged at 15,000× *g* for 30 s at 4 °C. To the supernatant and conditioned medium was added 66% metaphosphoric acid (10 µL/200 µL supernatant), and this solution was then incubated first at 4 °C for 30 min and then with 22 mg/mL dithioerythritol (10 µL/200 µL supernatant; MP Biomedicals, LLC, Illkirch, France) at 4 °C for 30 min. The supernatant was centrifuged and filtered for a later analysis. The levels of APPS and its metabolites were measured with HPLC equipped with a Shodex Asahipak NH2P-50 4E column. The separation conditions were as follows: detection wavelength, 245 nm; mobile phase, 60 mM H_3_PO_4_/acetonitrile (20/80) (Figure 3C).

### 2.5. Measurement of AA Content in Sod1-decifient Cells

**Figure 4:**
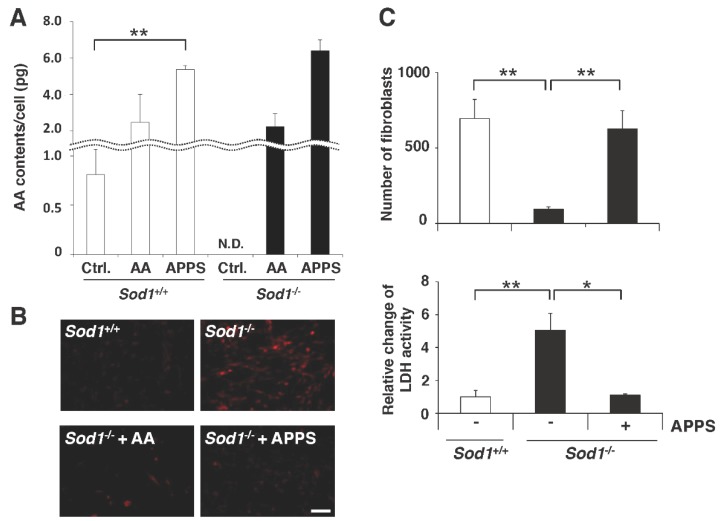
APPS elevates the cellular AA levels and attenuates cellular damage in skin cells. (**A**) Intracellular AA contents in *Sod1*^+/+^ and *Sod1*^−/−^ cells treated with 10 µM AA or 10 µM APPS for 6 h; (**B**) For the measurement of intracellular reactive oxygen species, cultured *Sod1*^+/+^ and *Sod1*^−/−^ cells treated with 10 µM AA or 10 µM APPS for 24 h were stained with dihydroethidium. The scale bar represents 100 µm; (**C**) The viability and proliferation of *Sod1*^+/+^ and *Sod1*^−/−^ cells with or without 10 µM APPS treatment for 96 h were analyzed. The lactate dehydrogenase activity in the conditioned medium used to culture the *Sod1*^+/+^ and *Sod1*^−/−^ skin cells for 96 h was measured. These data represent the mean ± SEM; * *p* < 0.05, ** *p* < 0.01.

*Sod1*^+/+^ and *Sod1*^−/−^ dermal fibroblasts were cultured in accordance with a previous description [17]. Cells were cultured for 6 h in culture medium with or without 10 µM AA and 10 µM APPS. The AA level was measured using the Vitamin C quantitative determination Kit (SHIMA Laboratories, Tokyo, Japan) as described above (Figure 4A).

### 2.6. Intracellular Reactive Oxygen Species

*Sod1*^+/+^ and *Sod1*^−/−^ dermal fibroblasts were cultured with 10 µM AA or 10 µM APPS for 24 h in 1% O_2_, followed by incubation under 20% O_2_ condition for 16 h to induce oxidative stress. After treatment, the fibroblasts were stained with 10 µM dihydroethidium (DHE) fluorescent probe (Life Technologies Corporation, Carlsbad, CA, USA) and 10 µM Hoechst 33342 (Merck Millipore, Darmstadt, Germany) for 20 min under 20% O_2_. The intracellular superoxide (O_2_^−^) generation was calculated as the DHE-positive area per nuclei number using fluorescent microscopy with the Leica Qwin V3 image software program (Leica Microsystems, Buffalo Grove, IL, USA).

### 2.7. Cell Viability and Proliferation Assay

*Sod1*^+/+^ and *Sod1*^−/−^ skin cells were cultured, and the number of cells was directly counted as described previously [17]. The collected medium was centrifuged at 400× *g* for 5 min at 4 °C, and the supernatant was used for the subsequent assays. The lactate dehydrogenase (LDH) level was measured using the LDH cytotoxicity assay kit (Cayman Chemical Company, Ann Arbor, MI, USA) in accordance with the manufacturer’s instructions.

### 2.8. Statistical Analyses

The statistical analyses were performed using Student’s *t-*test for comparisons between two groups and Tukey’s test for comparisons among three groups. Differences between the data were considered significant when the p values were less than 0.05. All data are expressed as the mean ± standard error of the mean (SEM).

## 3. Results

### 3.1. APPS Positively Increases the Intracellular AA Contents in an AA Transporter-Independent Manner

AA has been largely used in cosmetics to maintain the skin function because of its beneficial effects, such as antioxidation and regulation of collagen biosynthesis. However, its high fragility as well as low liposolubility limit its penetration into the skin and physiological action. A number of AA derivatives have been developed to overcome these disadvantages [10,11]. One such derivative, APPS, was generated through the conjugation of a phosphate group and a long hydrophobic chain (Figure 1).

In order to evaluate the permeability of ΑA, we treated human skin cells with AA or APPS and biochemically measured the cellular AA contents (Figure 2A). APPS treatment for 1 h significantly increased the cellular AA levels by 4.1-fold compared to the control skin, whereas AA treatment increased them only by 2.3-fold (Figure 2A). AA is usually transported into cells through AA transporters, such as SVCTs and GLUTs [18,19]. To investigate the transporter utilization of APPS and AA, we pre-treated human skin cells with PMA and glucose as AA transport inhibitors. As shown in Figure 2B, PMA and glucose markedly inhibited the uptake of AA with only AA addition alone. In contrast, APPS treatment sustained high levels of cellular AA in fibroblasts in the presence of both PMA and glucose (Figure 2B). These results demonstrated that APPS supplementation effectively and stably enhanced the intracellular AA contents in an AA transporter-independent manner.

### 3.2. Topical APPS is Effectively Converted to AA in Skin Cells

Next, to investigate the conversion mechanism of APPS to AA in skin cells, we incubated APPS in homogenates of human keratinocytes and monitored the dynamics of APPS and other metabolites, including AA, l-ascorbyl 6-palmitate (A6Pal), and sodium ascorbyl 2-phosphate (APS) (Figure 1). As expected, the APPS contents were rapidly reduced at 2 h after incubation (Figure 3A). In contrast, the contents of A6Pal, a metabolite with phosphate group cleavage, were increased at 2 h after incubation and gradually decreased until 8 h (Figure 3A). Concomitantly, the AA contents were gradually increased in a time-dependent manner (Figure 3A). Interestingly, the APS contents were not altered in the homogenates (Figure 3A). Indeed, potent phosphatase and esterase activities have been detected in human as well as rodent skin tissue [20,21,22,23]. Taken together, these present and previous findings suggest that endogenous phosphatases first cleave the phosphate group of APPS followed by intrinsic esterases to release the palmitate group of A6Pal, resulting in the production of AA in the conversion process.

To estimate the permeability of APPS, we applied APPS to our human epidermis models, which consist of a stratum corneum layer on keratinocyte culture (Figure 3B). Skin tissues without APPS treatment possessed AA contents below detection limit. When we treated the model with APPS for 24 h, the AA contents in the tissue was significantly increased in a dose-dependent manner (Figure 3C), suggesting that APPS was converted to AA in the tissue. Furthermore, the AA contents in the conditioned medium at the bottom of the dish were also significantly increased in cases of high-dose treatment (Figure 3C). These results indicated that APPS is effectively converted to AA and transferred into skin cells.

### 3.3. APPS Attenuates Cellular Oxidative Damage in Skin

SOD1, a major antioxidant enzyme in cytoplasm, plays an important role in maintaining the cellular redox balance. SOD1 loss significantly exhibited low viability associated with enhanced intracellular reactive oxygen species and cellular damage [24,25,26,27,28,29,30,31,32]. Interestingly, we failed to detect trace levels of cellular AA in *Sod1*^−/−^ cells, indicating impairment of the AA-glutathione cycle and redox balance (Figure 4A). APPS and AA treatment enhanced the cellular AA level in both *Sod1*^−/−^ and *Sod1*^+/+^ skin cells (Figure 4A). Pre-treatment with APPS and AA completely suppressed the O_2_^−^ generation in *Sod1*^−/−^ cells, resulting in production at the same level as in the *Sod1*^+/+^ cells (Figure 4B). APPS treatment also improved the viability and promoted proliferation associated with the suppression of cellular damage (Figure 4C). These findings showed that APPS ameliorates cellular damage by increasing the cellular AA level in damaged skin cells.

## 4. Discussion

### 4.1. APPS is Effectively Converted to AA by Cellular Convertases, Resulting in AA Transport into the Cytoplasm of Skin Cells in an AA Transporter-Independent Manner

Since skin innately includes relatively high levels of AA (approximately 50-fold that of plasma) [1,2], the skin AA level generally depends on and is maintained by AA transport activity rather than concentration-directed diffusion. In the present study, we showed that APPS treatment effectively supplied AA to the cytoplasm in skin cells compared to AA treatment in vitro (Figure 2A). APPS, but not AA, significantly increased the intracellular AA level even though both SVCTs and GLUTs were inhibited (Figure 2B). This preferential capacity of APPS is due to an AA transporter-independent action. As shown in Figure 3A, the addition of APPS to cell lysate rapidly increased the content of A6Pal, but not APS, and then gradually increased the AA content. We also found that APPS efficiently penetrated and converted AA in epidermal cells, leading to passed through AA in the conditioned medium in a three-dimensional epidermis model (Figure 3C). Since skin cells possess high phosphatase and esterase activity [20,21,22,23], endogenous cellular convertases can cleave the phosphate and palmitate groups of APPS.

Glatz et al. reported that fatty acids, including palmitate, can directly traverse the plasma membrane and that albumin proteins located at the outer cell surface may play an additional role in the delivery of fatty acids into the cytoplasm in cells [33,34]. These multiple transport mechanisms of fatty acid may help facilitate the permeability of skin cells to APPS, leading to its conversion to AA by endogenous cellular enzymes, such as phosphatases and esterases.

### 4.2. APPS Improves the AA Level and Skin Function by Regulating Redox Balance

In the present study, in vitro experiments showed that the supply of AA by APPS treatment effectively increased the cellular AA level and suppressed the O_2_^−^ generation associated with improved viability in *Sod1*^−/−^ cells, resulting in physiological redox level (Figure 4A,B). We also showed that treatment with APPS significantly promoted the proliferation and migration of *Sod1*^−/−^ skin cells associated with the suppression of LDH activity (Figure 4C). Du et al. reported that APPS treatment improved viability of PC12 cells treated with hydrogen peroxide [12], suggesting that APPS treatment protect cells from various types of oxidative damage. AA highly reacts with oxygen and O_2_^−^, resulting in oxidized AA forms such as mono-DHAA and DHAA [35,36]. Mono-DHAA and DHAA serve as AA radicals to capture electrons and can also be recycled back into AA by direct reduction in the AA-glutathione cycle [37]. Under highly oxidative conditions, mono-DHAA and DHAA are further degraded via hydrolysis or oxidation to 2,3-diketogulonic acid with no AA potency [38]. These results suggest that AA supplementation by APPS may increase AA recycling via these systems, resulting in improvement in the redox balance in damaged skin, such as under conditions of *Sod1* deficiency.

Long chain fatty acids, including palmitate, are required for the lipid synthesis in skin to maintain tissue homeostasis [39]. Kim et al. reported that ultraviolet (UV) irradiation and aging stress caused a reduction in the contents of palmitate, eicosatrienoic acid, and other fatty acid in skin [40]. Treatment with eicosatrienoic acid downregulated the expression of MMP1 in human keratinocytes irradiated by UV [40]. Palmitoleic acid, metabolites from palmitate, also inhibited the gene expression of *Mmp9* and RANKL-induced NF-κB activation in murine macrophages [41]. These results suggest that fatty acid supplementation to the skin may act as a regulator of skin homeostasis. In this context, palmitate cleaved from APPS in the skin might also protect skin cells from exogenous insults, such as pro-oxidants and UV.

### 4.3. Topical Application of APPS for Damaged Skin

The SVCT function is obligatorily dependent on a favorable inward gradient for Na^+^, which in turn is sustained by the continuous extrusion of Na^+^ by ATP-dependent Na^+^/K^+^-ATPase [42,43]. Indeed, the replacement of Na^+^ with K^+^, Li^+^, or choline almost completely abolishes the AA uptake [42,44]. Furthermore, SVCT2 is modulated by Ca^2+^ and Mg^2+^ ions, which switch the transporter from an inactive to an active form [45]. We previously reported that *Sod1* deficiency induced age-related skin atrophy and a reduction in the AA contents in skin [25]. We also provided evidence that *Sod1*^−/−^ skin cells showed aberrantly increased intracellular Ca^2+^ levels (data not shown) and loss of mitochondrial membrane potential associated with ATP depletion [46], indicating alteration of intracellular Ca^2+^ and ATP utilization in skin. We previously demonstrated that the topical treatment of APPS completely cured atrophy and oxidative damage in *Sod1*^−/−^ skins [17,27]. Taken together, these findings also implied that APPS thus appears to be useful for the supply of AA to the skin and also for the mitigation of oxidative damage via penetration mechanisms independent from AA transporters. Topical treatment of APPS is a beneficial strategy for supplying AA and improving the physiology of damaged skin.
